# Systematic Pelvic and Para-Aortic Lymphadenectomy During Fertility-Sparing Surgery in Patients With Early-Stage Epithelial Ovarian Cancer: A Retrospective Study

**DOI:** 10.3389/fonc.2022.913103

**Published:** 2022-06-17

**Authors:** Tingting Li, Ya Liu, Sixia Xie, Hongjing Wang

**Affiliations:** ^1^ Department of Gynecology and Obstetrics, West China Second University Hospital, Sichuan University, Chengdu, China; ^2^ Key Laboratory of Birth Defects and Related Diseases of Women and Children, Sichuan University, Ministry of Education, Chengdu, China

**Keywords:** systematic lymphadenectomy, early-stage, epithelial ovarian cancer, fertility-sparing surgery, survival

## Abstract

**Objective:**

The implication of pelvic and para-aortic lymphadenectomy in surgical staging of early-stage epithelial ovarian cancer (eEOC) is still debated. Limited data are available about systematic lymphadenectomy during fertility-sparing surgery (FSS) in patients with eEOC.

**Methods:**

The medical records of 38 patients with FIGO stage I EOC and below 40 years who underwent FSS at our hospital between January 2003 and December 2018 were retrospectively reviewed.

**Results:**

Among them, 18 patients (47.4%) underwent comprehensive lymphadenectomy, 11 patients (28.9%) underwent lymph node sampling, and 9 patients (23.7%) did not undergo lymphadenectomy. There was no statically significant difference in age, histology, grade, surgical approach, chemotherapy, and gestation among the three groups. With a median follow-up of 52.5 months (range: 24–153), three patients (7.9%) with FIGO stage IC EOC developed tumor recurrence. In these patients, progress-free survival (PFS) was 92.1%, and overall survival (OS) was 94.7%. No significant difference in the OS. Three patients had among all the patients, 15 patients (39.5%) had gestation after treatment, and 23 patients (60.5%) did not have gestation after treatment.

**Conclusion:**

The number of lymph nodes removed did not significantly affect survival eEOC with FSS. Systematic pelvic and para-aortic lymphadenectomy could not be performed for mucious eEOC patients with FSS if intraoperative freezing in confirmed and no suspicious lymph nodes are found. A better understanding of sentinel lymph node biopsy may help to identify whether the patient requires FSS.

## Introduction

EOC is the most lethal gynecologic malignancy and one of the leading causes of cancer-related death in women worldwide (1), with 21,750 new cases and 13,940 deaths recorded in 2020 in the United States (2). Although EOC predominantly occurs in older women, around 14% of EOC is diagnosed in women who are 40 years old or lesser who may not have completed childbearing (3). One important dilemma in managing such patients with EOC is preservation of reproductive function. Because conventional surgical excision for EOC causes infertility, fertility-sparing surgery (FSS) is a suitable alternative. According to the National Comprehensive Cancer Network (NCCN, 2022), FSS is recommended to patients with early-stage invasive epithelial tumors (4). The standard procedure of FSS is considered as unilateral saplingo-oophorectomy, comprehensive surgical staging surgery for stage IA unilateral EOC. For those with bilateral stage IB EOC, preserving the uterus and comprehensive surgical staging can be considered. The use of systematic lymphadenectomy is an area of controversy, systematic lymphadenectomy improved detection of positive lymph nodes and OS in early-stage disease but not improve PFS (4). In many reports regarding FSS for treating EOC, lymphadenectomy was optional. However, comprehensive surgical staging is necessary for patients who need FSS, since CT has poor sensitivity for the low-volume nodal disease. The risk of occult pelvic and/or para-aortic lymph node metastases in the apparent early-stage FIGO stage I and II (5) EOC ranges from 6.1% to 29.6%, with a mean incidence of 14.2% (6, 7). For patients with presumed early stage, systematic pelvic and para-aortic lymphadenectomy may prevent unnecessary postoperative chemotherapy and has a proven prognostic value for eEOCs (6). Although knowing the lymph node status is necessary for the accurate staging diagnosis during the surgical procedure, determining the status accurately is difficult because of several serious issues for young women. Lymphadenectomy is the major surgical procedure associated with other morbidities such as lymphedema, lymphocyst, ileus, blood loss, nerve or vascular injury, blood transfusion, prolonged hospital stays, and high treatment costs (8, 9). The role of lymphadenectomy for eEOC patients who wish to undergo FSS remains controversial. Only limited and conflicting data assessing the role of systematic pelvic and para-aortic lymphadenectomy during FSS in EOC are available (7–10). Therefore, in this study, we aimed to understand the implication of pelvic and para-aortic lymphadenectomy during FSS in patients with eEOC.

## Methods

### Study Population

Patients with FIGO stage I EOC and below 40 years, who had information for the extent of pelvic and para-aortic lymphadenectomy at West China Second Hospital of Sichuan University between January 2003 and December 2018, were retrieved for this retrospective study. We examined the 4 most common histologic subtypes of EOC: serous, endometriod, clear cell, and mucinous, some of the patients had more than two of these types were classified as mixed epithelial. Exclusion criteria of the study were women with FIGO stage II-IV disease, non-epithelial histology types, epithelial ovarian cancer but not the aforementioned 4 histologic types, lack of information for pelvic and para-aortic lymphadenectomy, borderline ovarian tumors, and metastatic tumors to the ovary. The approval of the Institutional review board was obtained to conduct this study. All patients provided written informed consent. All specimens of surgery were reviewed by our expert pathologist to confirm the histological diagnosis. The patients included in the analysis were those who received FSS and wanted to retain the ability to bear a child.

FSS was performed by laparotomy and laparoscopy. The standard process included peritoneal lavage for fluid or ascites cytology, unilateral salpingo-oophorectomy, omentectomy, pelvic and/or abdominal para-aortic lymph node dissection, removal of visible lesions, and biopsy of suspicious sites (11). Pelvic lymphadenectomy includes the external and internal iliac, superficial and deep common iliac, superficial and deep obturator, and presacral nodes. Aortic lymphadenectomy starts at the aortic bifurcation up to the renal vessels by removing superficial and deep intercavoaortic, precaval, paracaval, preaortic, and paraaortic nodes (12). In our study, because of the controversy regarding lymphadenectomy and the patients’ willingness to surgery, some of the patients underwent lymphadenectomy, while others did not undergo lymphadenectomy. According to the number of lymph nodes, comprehensive lymphadenectomy was defined as ≥20, while lymph node sampling was defined as 1–19, histologically confirmed pelvic and para-aortic lymph nodes were removed (13). Because the patients mostly underwent pelvic lymphadenectomy or/and para-aortic lymphadenectomy, our patients who underwent FSS were divided into three groups based on the number of lymph nodes, including the comprehensive lymphadenectomy group (lymph nodes ≥20), the lymph node sampling group (lymph nodes from 1 to 19), and the non-lymphadenectomy group. After surgery, adjuvant chemotherapy was recommended for the patients at FIGO stage IC or more, with grade 2–3 tumor or showing clear cell histology. Most patients after fertility-sparing surgery received 1-7 cycles of carboplatin/cisplatin-based chemotherapy, but some patients refused chemotherapy due to desire to get pregnant early or personal reasons.

### Data Collection

The clinicopathological data of the patients were obtained from medical charts. A follow-up examination was recommended every three months for the first two years and every six months for the next two years, and then once a year. Disease-free survival (DFS), overall survival (OS), menstrual conditions, fertility, and gestational outcomes were evaluated. Missing data were collected through multiple telephone interviews during follow-up. DFS was measured from the start of the treatment until the date of detection of recurrence. OS was measured from the start of the treatment until the date of death, or for living patients, to the date of the last follow-up (14).

### Statistical Analysis

The characteristics of the clinicopathologic categorical variables in women who did or did not undergo lymphadenectomy were compared by performing Person’s *χ* ^2^ test or Fisher’s exact test, while measurement data were assessed by performing Student’s *t*-tests. Univariate analyses and differences in survival were assessed by performing the log-rank test. Kaplan-Meier curves were plotted to examine survival based on whether lymphadenectomy was performed. Only those variables were included in the model whose difference between or among the groups had a *p*-value below 0.05. The SPSS 24.0 software was used to conduct statistical analyses.

## Results

### Characteristics of the Patients

The characteristics of the patients are summarized in **Table 1**. 38 patients with eEOC who underwent FSS were included in this study. Of these patients, 18 patients (47.4%) underwent comprehensive lymphadenectomy, 11 patients (28.9%) underwent lymph node sampling, and nine patients (23.7%) did not undergo lymphadenectomy. Among the 29 patients who underwent retroperitoneal staging, the median number of lymph nodes removed was 22 (range: 13–36), the median number of pelvic and para-aortic lymph nodes was 19 (range: 8–33) and 2 (range: 0–8), respectively. No evidence of metastatic disease was found. The median age at diagnosis was 26 years (range: 14–39). The most common histology type was mucinous (n = 27, 71.1%), followed by endometroid (n = 6, 15.8%), clear cell (n = 2, 5.3%), mixed epithelial (n = 2, 5.3%), and serous (n = 1, 2.6%). The FIGO stage was IA in 15 patients (39.5%) and IC in 23 patients (60.5%). There were no patients in the IB stage. A total of 29 patients (76.3%) had grade 1 tumors, whereas five patients (13.2%) had grade 2 tumors, and four patients (10.5%) had grade 3 tumors. Sixteen patients (42.1%) underwent one surgery, while 22 patients (57.9%) underwent two surgeries. These patients who underwent two surgeries were often due to incomplete staging of the previous surgery or the decision to expand the surgical scope after the initial operation. Complete staging surgery was performed in 29 patients, and only nine patients did not undergo complete staging surgery. Regarding the surgical procedure at the time of lymphadenectomy, because of the size of tumors, we found that 26 patients (68.4%) were operated by laparotomy to avoid tumor rupture, and 12 patients (31.6%) were operated by laparoscopy. 28 (73.7%) patients received postoperation adjuvant chemotherapy, while 10 patients (26.3%) did not receive chemotherapy. Among all the patients, 15 patients (39.5%) had gestation after treatment, and 23 patients (60.5%) did not have gestation after treatment.

**Table 1:** The clinical oncological data of all 38 patients with eEOC.

Factors	All cases(n = 38)	Comprehensive Lymphadenectomy (n = 18)	Lymph node sampling(n = 11)	No lymphadenectomy(n = 9)	*p* values
Age at surgery, years Median (range)	26 (14–39)	26 (14–36)	24 (15–34)	28 (21–39)	0.383
Histology					0.308
Serous	1 (2.6%)	1 (5.6%)	0 (0.0%)	0 (0.0%)	
Mucinous	27 (71.1%)	15 (83.3%)	7 (63.6%)	5 (55.6%)	
Endometriod	6 (15.8%)	1 (5.6%)	2 (18.2%)	3 (33.3%)	
Clear cell	2 (5.3%)	0 (0.0%)	1 (9.1%)	1 (11.1%)	
Mixed epithelial	2 (5.3%)	1 (5.6%)	1 (9.1%)	0 (0.0%)	
FIGO stage					< 0.001
IA	15 (39.5%)	5 (27.8%)	4 (36.4%)	6 (66.7%)	
IC	23 (60.5%)	13 (72.2%)	7 (63.7%)	3 (33.3%)	
Grade					0.342
G1	29 (76.3%)	15 (83.3%)	7 (63.6%)	7 (77.8%)	
G2	5 (13.2%)	2 (11.1%)	3 (27.3%)	0 (0.0%)	
G3	4 (10.5%)	1 (5.6%)	1 (9.1%)	2 (22.2%)	
Total number of lymph nodes removed, median, (range)	22 (13–36)	26 (20–36)	17 (13–19)		< 0.001
Median number of pelvic lymph nodes removed, (range)	19 (8–33)	23 (17–33)	15 (8–18)		< 0.001
Median number of para-aortic lymph nodes removed, (range)	2 (0–8)	3 (0–8)	1 (0–7)		0.087
Number of surgeries					< 0.001
One	16 (42.1%)	4 (22.2%)	3 (27.3%)	9 (100.0%)	
Two (re-staging)	22 (57.9%)	14 (77.8%)	8 (72.7%)	0 (0.0%)	
Surgical approach at time of lymphadenectomy					0.666
Laparotomy	26 (68.4%)	13 (72.2%)	8 (72.7%)	5 (55.6%)	
Laparoscopy	12 (31.6%)	5 (27.8%)	3 (27.3%)	4 (44.4%)	
Complete staging surgery					< 0.001
Yes	29 (76.3%)	18 (100.0%)	11 (100.0%)	0 (0.0%)	
No	9 (23.7%)	0 (0.0%)	0 (0.0%)	9 (100.0%)	
Chemotherapy					0.564
Yes	28 (73.7%)	12 (66.7%)	8 (72.7%)	8 (88.9%)	
No	10 (26.3%)	6 (33.3%)	3 (27.3%)	1 (11.1%)	
Gestation after treatment					0.914
Yes	15 (39.5%)	7 (38.9%)	5 (45.5%)	3 (33.3%)	
No	23 (60.5%)	11 (61.1%)	6 (54.5%)	6 (66.7%)	

We found that the proportion of patients in the comprehensive lymphadenectomy group or lymph node sampling group was higher than that with no lymphadenectomy (*p* < 0.001). Patients with comprehensive lymphadenectomy or lymph node sampling had higher numbers of removed lymph nodes (*p* < 0.001) and higher numbers of removed pelvic lymph nodes (*p* < 0.001). The proportion of patients who underwent re-staging surgery was significantly higher in the comprehensive lymphadenectomy group or the lymph node sampling group than the proportion of patients with no lymphadenectomy (*p* < 0.001). The patients with comprehensive lymphadenectomy or lymph node sampling underwent complete surgeries, while the patients with no lymphadenectomy did not undergo complete surgery (*p* < 0.001). There was no statistically significant difference in age (*p* = 0.383), histology (*p* = 0.308), grade (*p* = –0.342), surgical approach (*p* = 0.666), chemotherapy (*p* = 0.564), and gestation (*p* = 0.914) among the patients in the three groups.

### Survival Analysis

**Figure 1:**
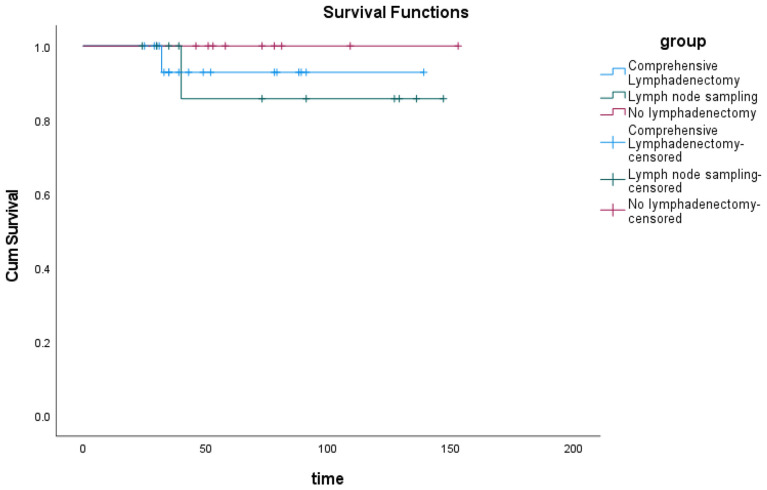
The overall survival of patients with eEOC who underwent comprehensive lymphadenectomy or lymph node sampling or did not undergo lymphadenectomy.

With a median follow-up of 52.5 months (range: 24–153), three patients (7.9%) developed tumor recurrence (**Table 2**). Three patients had FIGO stage IC epithelial ovarian carcinoma; their histology was mucinous, mixed epithelial (endometrioid and mucinous), and mucinous, respectively. One patient was G3; the others were G1. Three patients underwent laparotomy and second re-staging surgery and chemotherapy. Two patients underwent comprehensive lymphadenectomy, and one patient underwent lymph node sampling. Two patients (5.3%) died due to tumor recurrence. The first one patient underwent four cycles of TP chemotherapy after second re-staging surgery and showed recurrence in the abdominal incision after 23 months, then she was treated with palliative surgery due to extensive recurrent lesion and underwent once cycle of TC and twice cycles of TP chemotherapy, consequently died of the disease after a follow-up of 32 months. The second patient underwent one intraperitoneal chemotherapy dominated by cisplatin after first surgery, and twice cycles of intraperitoneal chemotherapy dominated by cisplatin after second re-staging surgery. She showed recurrence in the ascending colon three months after the second laparotomy re-staging surgery, and she underwent colon surgery and eight cycles of TP chemotherapy. She was still alive without signs of the disease. The third patient underwent one cycle of Docetaxel combined with carboplatin of chemotherapy after first surgery and underwent three cycles of TP chemotherapy after second re-staging surgery. She showed recurrence in the sigmoid colon 24 months after the second laparotomy re-staging surgery, she was treated with surgery and six cycles of TP chemotherapy. One years later, she died of the disease after a follow-up of 40 months. All of three patients were treated with incomplete staging surgery out of our hospital, we hypothesized that there might have been a tumor rupture during surgery. The site of their recurrence was the pelvic cavity, therefore, recurrence may be due to intraperitoneal tumor spread. In these patients, PFS was 92.1%, and OS was 94.7%. No significant difference in the overall survival was found when grouping based on the number of lymph nodes removed, as comprehensive lymphadenectomy, lymph node sampling, and no lymphadenectomy (p = 0.594) (**Figure 1**).

**Table 2:** The information of the patients who developed recurrence.

Case	Age at surgery	FIGO stage	Histology	Grade	Type of lymphadenectomy	Recurrence site	Disease-free survival	Overall survival	Outcome
1	27	IC	Mucinous	G3	Comprehensive Lymphadenectomy	Abdominal incision	23	32	DOD
2	36	IC	Mixed epithelial(Endometrioid and Mucinous)	G1	Comprehensive Lymphadenectomy	Colon	3	30	NED
3	24	IC	Mucinous	G1	Lymph node sampling	Colon	24	40	DOD

DOD, died of disease; NED, no evidence of disease.

## Discussion

The prognostic value of the involvement of lymph nodes is confirmed by the FIGO stage. The risk of occult pelvic and/or para-aortic lymph node metastases in apparent early-stage (FIGO stage I and II (5)) epithelial ovarian cancer ranges from 6.1% to 29.6%, with a mean incidence of 14.2% (6, 7). Recently, the ESMO-ESGO consensus conference on ovarian cancer recommended that lymph node dissection for re-staging purposes may be avoided if the nodal status does not alter the patient’s condition (15).

We evaluated the impact of systematic pelvic and para-aortic lymphadenectomy during FSS in patients with eEOC. According to the number of lymph nodes, our patients who underwent FSS were divided into three groups. Our result suggested that the overall survival of the patients in the three groups was similar. The number of removed lymph nodes did not cause any significant survival difference in patients with eEOC who underwent FSS. Several studies have evaluated the impact of systematic pelvic and para-aortic lymphadenectomy on eEOC (13, 16–21). Many studies demonstrated the prognostic value of comprehensive lymphadenectomy in eEOC (16–20). However, the survival implication of systematic pelvic and para-aortic lymphadenectomy in surgical staging of eEOC is still under debate. Gao et al. (16) compared the five-year survival rates and showed that lymphadenectomy could improve the five-year overall survival rate of patients with advanced-stage EOC but not that of patients with early-stage EOC or residual tumors (≤2 cm). Meta-analyses that included retrospective or observational studies have reported that systematic lymphadenectomy improves OS in patients with early stage disease, even though it does not improve PFS (22).The only randomized controlled trial on the therapeutic role of lymphadenectomy in eEOC, conducted by Maggioni et al. (21), showed similar disease-free survival and overall survival between systematic lymphadenectomy and lymph node sampling, with a hazard ratio of 0.72 and 0.85, respectively, in favor of systematic lymphadenectomy. Bizzarri et al. (13) found that there was no difference in the five-year overall survival in apparent eEOC patients who underwent and did not undergo lymphadenectomy. The results are consistent with that of our study. Those results suggested that the number of lymph nodes removed does not significantly affect eEOC, which is probably because the rate of lymph node metastasis is low in apparent eEOC.

The histology and degree of differentiation have significant impact on the survival of eEOC patients with or without systematic lymphadenectomy. Ceballos et al. (17) reported, based on a study of the early-stage mucinous ovarian cancer, that complete surgical staging with lymph node dissection did not affect recurrence, disease-free period, and overall survival. Giorgio et al. (18) suggested that high-grade serous and bilateral eEOC are at a high risk of harboring diseases in the lymphatic tissues of both pelvic and para-aortic areas. They argued that high-grade serous and bilateral early-stage epithelial ovarian cancer patients require comprehensive retroperitoneal staging. Yuji et al. (19) showed that the number of removed lymph nodes (≥35) was an independent predictor for improved recurrence-free survival in stage I ovarian clear cell carcinoma. Yuji et al. (19) suggested that sufficient lymphadenectomy may improve the prognosis for stage I ovarian clear cell carcinoma. In our study, 71.1% of the patients had mucinous EOC, 76.3% of the patients had low-grade EOC. Although, our study suggested that histology and degree of differentiation did not affect the survival of eEOC, 71.1% of our patients were mucinous and 76.3% were G1. At the end on the last NCCN guideline (4), we find that if mucinous histology is confirmed by intraoperative frozen section analysis and there are no suspicious lymph nodes is possible to consider omitting lymphadenectomy. Also, ESMO-ESGO consensus conference on ovarian cancer recommended that lymph node dissection for re-staging purposed may be avoided if the nodal status does not alter patients’s management. So, systematic pelvic and para-aortic lymphadenectomy could not be performed for mucious eEOC patients with FSS if intraoperative freezing in confirmed and no suspicious lymph nodes are found.

Systematic pelvic and para-aortic lymphadenectomy is performed to identify retroperitoneal metastasis and avoid missing occult tumors. Additionally, there are several serious issues for young women who undergo systematic pelvic and para-aortic lymphadenectomies, such as cosmetic problems due to extension of the abdominal wound scar and the possibility of tubal infertility due to the extensive range of adhesion, longer operation time and higher proportion of patients requiring blood transfusions (10, 21). It can increase the rate of severe postoperative complications and delaying the time of chemotherapy administration (13). Similar to the results of the previous studies, we found that systematic pelvic and para-aortic lymphadenectomy cannot significantly improve the overall survival of eEOC. In our series, especially in those 29 patients who underwent lymphadenectomy or lymph node sampling, no positive lymph nodes were found. Since these patients are studied with CT scan dan therefore apparently early stage, we expect a low rate of positive lymph nodes, so if the number of removed lymph nodes does not alter the overall survival in these patients, we could think of a qualitative approach. In this context, the potential role of sentinel lymph node biopsy may be of interest (23), as sentinel lymph node biopsy is widely used in early-stage cervical cancer and endometrial cancer (24). If sentinel lymph node biopsy can be performed, it can improve the detection of positive lymph node by qualitative approach rather than the quantity of lymphadenectomy, it can determine whether the patient can undergo fertility preservation by FIGO staging earlier.

This study has two limitations. First, this study is retrospective. Considerably old cased were included and the scope of lymphadenectomy in old cases was different from that in the current cases. Secondly, this study was conducted in a single institution. Accordingly, the low number of cases was small compared with those in multicenter studies, and heterogeneity of the sample as well as of the adjuvant treatment were a limitation. However, this condition had an advantage that the lymphadenectomy procedure was uniformed because it was performed at a single institution.

## Conclusions

In conclusion, we demonstrated the implication of pelvic and para-aortic lymphadenectomy during FSS of patients with eEOC. We found that the number of lymph nodes removed did not significantly affect survival eEOC with FSS. Systematic pelvic and para-aortic lymphadenectomy could not be performed for mucious eEOC patients with FSS if intraoperative freezing in confirmed and no suspicious lymph nodes are found. Sentinel lymph node biopsy might play an important role in eEOC patients undergoing FSS in the future.

## Data Availability Statement

The original contributions presented in the study are included in the article/supplementary material. Further inquiries can be directed to the corresponding author.

## Ethics Statement

The studies involving human participants were reviewed and approved by Ethics Committee of West China Second Hospital, Sichuan University. Written informed consent to participate in this study was provided by the participants’ legal guardian/next of kin.

## Author Contributions

TL and YL collect and analyzed the data and wrote the manuscript. SX and HW made substantial contributions planning this work. TL and HW substantially revised the work and manuscript. All authors were involved in drafting and revising the manuscript, and all authors read and approved the final manuscript. All authors contributed to the article and approved the submitted version.

## Author Disclaimer

All claims expressed in this article are solely those of the authors and do not necessarily represent those of their affiliated organizations, or those of the publisher, the editors and the reviewers. Any product that may be evaluated in this article, or claim that may be made by its manufacturer, is not guaranteed or endorsed by the publisher.

## Conflict of Interest

The authors declare that the research was conducted in the absence of any commercial or financial relationships that could be construed as a potential conflict of interest.

## Publisher’s Note

All claims expressed in this article are solely those of the authors and do not necessarily represent those of their affiliated organizations, or those of the publisher, the editors and the reviewers. Any product that may be evaluated in this article, or claim that may be made by its manufacturer, is not guaranteed or endorsed by the publisher.

## Abbreviations

FIGO, International Federation of Obstetrics and Gynecology; EOC, epithelial ovarian cancer; FSS, fertility sparing surgery; NCCN, National Comprehensive Cancer Network; DFS, disease-free survival; OS, overall survival; ESMO-ESGO, European Society for Medical Oncology-European Society of Gynecological Oncology.
